# Physicochemical Characterization and Optimized Ultrasound-Assisted Extraction of Bioactive Compounds from Teff (*Eragrostis tef*): Phenolic Profile, Antioxidant Activity, Prebiotic Potential, and Phytic Acid Degradation

**DOI:** 10.3390/foods15152717

**Published:** 2026-08-01

**Authors:** Boyiza Samson Abebe, Iuliana Aprodu, Daniela Ionela Istrati, Nicoleta Balan, Aida Mihaela Vasile, Mihaela Cotârleț, Nicoleta Stănciuc, Camelia Vizireanu

**Affiliations:** Faculty of Food Science and Engineering, Dunarea de Jos University of Galati, 111 Domneasca Street, 800201 Galati, Romania; samson.abebe@ugal.ro (B.S.A.); iuliana.aprodu@ugal.ro (I.A.); nicoleta.balan@ugal.ro (N.B.); aidamihaela48@gmail.com (A.M.V.); mihaela.cotarlet@ugal.ro (M.C.); nicoleta.stanciuc@ugal.ro (N.S.); camelia.vizireanu@ugal.ro (C.V.)

**Keywords:** teff (*Eragrostis tef*), ultrasound-assisted extraction, Box–Behnken design, HPLC profiling, phenolic compounds, antioxidant activity, prebiotic potential, phytic acid degradation

## Abstract

Teff (*Eragrostis tef*) is a gluten-free cereal and a valuable source for functional food. This study evaluated the physicochemical characteristics of teff flours and optimized ultrasound-assisted extraction of bioactive compounds. Ultrasonic-assisted extraction (UAE) was optimized using a Box–Behnken design (BBD), and the best extraction conditions were 50% ethanol, 60 °C, and 45 min, yielding 49.6 ± 0.028 mg gallic acid equivalents (GAE)/100 g dry weight (DW). High-performance liquid chromatography (HPLC) analysis revealed an abundant phenolic profile with compounds including epigallocatechin (2960.30 µg/g), epicatechin (113.55 µg/g), hesperidin (163.64 µg/g), and ferulic acid (41.79 µg/g). Prebiotic potential testing with *Lactobacillus plantarum* MIUG BL21 at 4 °C over 21 days indicated a concentration-dependent decrease in viability: at 5 mg/mL, mean counts decreased from 4.721 ± 0.049 to 2.349 ± 0.048 log colony-forming units (CFU)/mL; at 2.5 mg/mL, from 3.396 ± 0.095 to 0.500 ± 0.198 log CFU/mL; and at 1 mg/mL, counts became undetectable. Phytic acid degradation in teff extracts followed first-order kinetics, with half-lives of 1504.55, 752.30, and 300.91 min at 50, 70, and 90 °C, respectively. The activation energy of 39.07 kJ/mol confirmed the temperature dependence of the process and indicated an effective reduction in phytic acid. In conclusion, teff extracts may represent promising functional ingredients with antioxidant potential, support for probiotic viability, and reduced antinutritional factors.

## 1. Introduction

Cereals are extremely versatile foodstuffs and are processed into a very wide range of traditional food and beverage products. Teff [*Eragrostis tef* (Zucc.) Trotter] is an annual crop in the Poaceae (grass) family with very tiny grains used primarily in Ethiopia to prepare a sour, fermented, pancake-like flatbread called injera [1,2]. Teff’s cultivation has spread beyond its native regions due to its nutritional benefits and gluten-free nature, making it increasingly popular in the global health food market. It is a highly nutritious grain widely consumed in Ethiopia and Eritrea and valued for its functional properties [3]. Compared with other cereals, teff is a gluten-free cereal rich in fiber, protein, and some essential nutrients [4]. Despite its nutritional and health benefits, teff has a higher phytic acid content (800–1000 mg/100 g), an antinutritional factor that can compromise the bioavailability of vitamins and minerals [5,6]. The grain has different varieties, including white and brown, each with distinct nutritional properties [7].

The comprehensive nutritional attributes of teff are particularly remarkable and provide relatively higher levels of essential amino acids, dietary fiber, and minerals such as calcium, copper, and zinc compared to other cereal grains consumed as whole-grain flours, such as sorghum, maize, barley, and wheat [3,8,9]. Additionally, teff contains a variety of bioactive compounds, including total phenolic content ranging across varieties between 1.2 and 2.5 mg GAE/g and flavonoids (1.4 mg QE/g), which have been linked to various health benefits, including antioxidant and anti-inflammatory properties [7,10]. These values provide a clearer scientific context when compared to studies by Koubová et al. [9], which reported TPC values as high as 11.47 mg GAE/g in red teff, and Gebru et al. [10] identified over 60 distinct bioactive compounds, including ferulic, protocatechuic, and p-coumaric acids [11]. These attributes provide essential baseline data for linking specific compositional qualities to biological activities and for optimizing processing protocols. Advanced extraction technologies, particularly ultrasonic-assisted extraction (UAE), offer significant advantages over conventional methods through enhanced mass transfer, reduced processing time, lower solvent consumption, and improved recovery of thermolabile bioactive compounds [12,13]. Consuming teff grain is strongly linked to a lower risk of chronic diseases such as cardiovascular disease, diabetes, and cancer [14,15]. Additionally, teff has prebiotic properties that can elevate gut health, making it a valuable dietary addition, mainly for individuals with specific dietary requirements. The dietary fiber fractions in teff, including arabinoxylans, β-glucans, and resistant starch, along with associated phenolic compounds, may work together to produce prebiotic effects through selective fermentation by beneficial gut bacteria and by influencing the intestinal environment [16,17].

High-performance liquid chromatography (HPLC) is the preferred analytical method for analyzing bioactive compounds in a variety of foods because it allows the separation, identification, and quantification of components in complex mixtures, such as teff extracts [18]. HPLC profiling of red teff extracts can be used to determine the types and amounts of prebiotic oligosaccharides, phenolic acids, flavonoids, and other intermediary metabolites that impact gut health. The HPLC profile serves as a fingerprint of the composition, providing the basis for quality control, standardization, and linking composition to biological activities [19]. By combining HPLC with extraction optimization, researchers can achieve targeted recoveries of specific compound classes, enabling the development of extraction methods that leverage knowledge of the chemical composition to guide their manufacture rather than relying solely on empirical trials.

Recent studies have highlighted the potential of teff for functional food development, owing to its bioactive peptides, which exhibit antioxidant and antihypertensive activities [20]. The crop’s high phenolic content, especially in the red variety, contributes to its antioxidant capacity, as demonstrated in both cell-free and cell-based models [10,21]. The differences among white, mixed, and red teff varieties further emphasize variability in bioactive composition and functional properties. This growing body of research highlights teff’s potential as an important part of health-promoting diets and its role in chronic disease prevention [7,21]. While the nutritional benefits of teff are well-documented, there remains a need to bridge the gap between optimized extraction protocols and the kinetics of antinutritional factors degradation. High-performance liquid chromatography (HPLC) serves as a vital analytical fingerprint in this regard, enabling the standardization of bioactive recovery and linking specific compositional qualities to health outcomes. Hence, the main objective of this study is to characterize teff by enhancing the extraction of its bioactive compounds, classifying these bioactive elements, exploring the degradation kinetics of phytic acid in teff, and assessing their functional properties.

## 2. Materials and Methods

### 2.1. Chemicals, Reagents, and Instruments

Sodium carbonate, sodium hydroxide, and Folin–Ciocalteu reagent were sourced from Remed Prodimpex S.R.L. (Bucharest, Romania); gallic acid, ethanol, and methanol of HPLC grade were purchased from Sigma-Aldrich, Steinheim, Germany; 2,2-Diphenyl-1-picrylhydrazyl (DPPH), 6-hydroxy-2,5,7,8-tetramethylchromane-2-carboxylic acid (Trolox), catechin, sodium acetate, sodium nitrite, quercetin, electric grinder (Heinner HCG-150SS; Bucharest, Romania), a Rotary Vacuum Concentrator with a cooling trap (RVC 218 CDplus model and CT02-50, respectively; Martin Christ, Osterode am Harz, Germany), Ultrasonic Bath (Mod. DU-32; ARGOLAB; Capri, Italy), and AlCl_3_ were also used. Ultrapure water (0.058 μS/cm) was obtained from a water purification system. A UV-Vis spectrophotometer (Cary 60, Agilent Technologies, Santa Clara, CA, USA) was used to measure the absorbance of prepared standards and sample solutions. A quartz cuvette with a 1 cm path length was used as the sample holder. A scientific ultrasonic cleaner (WUC-D10H, Wertheim, Germany) was used for sample extraction, and a centrifuge (Universal 320R; Hettich, Germany) was used to centrifuge the extracts. Deionized water was used throughout the experiment. The probiotic strain *Lactobacillus plantarum* MIUG BL21 was provided by Chr. Hansen, Hørsholm, Denmark, as a freeze-dried commercial starter.

### 2.2. Sample Preparation

Two kilograms of red teff (*Eragrostis tef*) were purchased (whole grain) from Paradisul Verde, Brasov, Romania, in May 2024, and the other mixed and white teff varieties were purchased from a local supermarket in Addis Ababa, Ethiopia. A 240 g portion of each dry teff sample was ground separately for 30 s using a Heinner HCG-150SS grinder (Heinner, Bucharest, Romania) to obtain flour. The resulting flours were transferred to separate, dry polyethylene containers and stored under dry conditions until further analysis.

### 2.3. Ultrasound-Assisted Extraction

Ultrasonication was used to assist the extraction of all teff components using an ultrasonic bath (Digital Ultrasonic Bath Model DU-32, manufactured by 131 ARGOLAB, Capri, Italy). The teff was ground to a powder prior to extraction, and 1 g of ground teff was transferred into a glass beaker and extracted with 9 mL of solvent mixture (1:9, *w*/*v*) consisting of 1 mL of acetic acid and 8 mL of 70% ethanol (1:8 *v*/*v*) for 45 min at 45 °C, using matching extraction methods. The extraction conditions were selected to optimize phenolic extraction while maintaining the structure and stability of all phenolics [22]. The ultrasound parameters were kept constant at 40 kHz and 100 W. To maintain a constant temperature, cold water was added to the ultrasonic bath. After extraction, the samples were centrifuged at 4153× *g* for 10 min at 4 °C (Universal 320R; Hettich, Germany), and the supernatants were concentrated using a rotary vacuum concentrator with a cooling trap (RVC 218 CDplus model and CT02-50, respectively; Martin Christ, Osterode am Harz, Germany). The residues were redissolved in methanol, filtered through 0.45 µm membranes, and analyzed by HPLC for quantifying bioactive compounds [23,24].

### 2.4. Experimental Design

To optimize and validate the extraction method using ethanol solvent, a Box–Behnken design (BBD) combined with three-level Response Surface Methodology (RSM) was used to optimize an ultrasound-assisted extraction (UAE) technique for bioactive compounds from teff flour [25,26]. Using this method, the extraction process systematically determined how various extraction parameters affected overall extraction efficiency and any potential interactions among them. The three response variables used to evaluate extraction performance were Total Phenolic Content (TPC), Total Flavonoid Content (TFC), and antioxidant activity determined by the 2,2-diphenyl-1-picrylhydrazyl (DPPH) radical scavenging method. The primary purpose of this experiment was to determine the optimum conditions for extracting the maximum amount of phenolic compounds while maintaining their biological activity.

The three independent variables were selected for optimization based on prior experiments and a relevant literature review. Ethanol concentration ranged from 50 to 90% (*v*/*v*), extraction time from 30 to 60 min, and extraction temperature from 30 °C to 60 °C [27,28]. These ranges were carefully chosen to establish adequate recovery of heat-sensitive bioactive compounds while lowering thermal degradation. The BBD resulted in 15 experimental runs, including three replicate runs at the center point (70% ethanol, 45 min, 45 °C) to estimate experimental error and assess method reproducibility. All experiments were conducted in a randomized order to reduce the impact of systematic errors and uncontrolled variations on the measurement responses. The levels and ranges of the independent variables used in the Box–Behnken design for optimizing the ultrasound-assisted extraction process are presented in Table 1.

The experimental data were modeled using a second-order polynomial regression model, expressed as follows:

*Y* = *βo* + *β*_1_*AX*_1_ + *β*_2_*BX*_2_ + *β*_3_*CX*_3_ + *β*_12_
*AB* + *β*_13_*AC* + *β*_23_*BC* + *β*_11_*A*^2^ + *β*_22_*B*^2^ + *β*_33_*C*^2^ (1)

where Y indicates the predicted response; A, B, and C are the coded independent variables [A—ethanol concentration (%, *v*/*v*); B—extraction time (min); and C—Temperature (°C)], β_0_ is the intercept; β_1_, β_2_, and β_3_ are the linear coefficients; β_11_, β_22_, and β_33_ are the quadratic coefficients; and β_12_, β_13_, and β_23_ are the interaction coefficients.

The regression coefficients were estimated using multiple regression analysis, and model adequacy was assessed using the coefficient of determination (R^2^), adjusted R^2^, and the lack-of-fit test. The significance of individual model terms was evaluated using analysis of variance (ANOVA) at the 5% significance level (α = 0.05), with *p*-values < 0.05 considered statistically significant.

Three-dimensional response surface plots and two-dimensional contour plots were produced to demonstrate the influence of independent variables on the extraction responses and to define the nature of their interconnections [29]. These visualizations helped identify the optimal extraction conditions. Numerical optimization was then performed using the desirability function approach to identify the combination of extraction parameters that simultaneously maximizes TPC, TFC, and antioxidant activity, thereby determining the compromise optimal conditions that meet all response criteria.

### 2.5. Determination of Total Phenolic Content (TPC) and Total Flavonoid Content (TFC)

The total phenolic content (TPC) was determined by using the Folin–Ciocalteu method, and the following experimental procedure was carried out according to Wani [30], with some modifications. A total of 200 μL of each sample extract was mixed with 15.8 mL of ultra-pure water and 1000 μL diluted Folin–Ciocalteu reagent, then combined with 3 mL of a 20% sodium carbonate solution (*w*/*v*), followed by a 1 h incubation in the dark at room temperature. After incubation, absorbance was measured at 756 nm using a spectrophotometer (Biochrom Libra 22 UV/Visible Spectrophotometer; 177 Holliston, MA, USA). Total phenolic content was assessed using gallic acid as the reference standard, and results were expressed in milligrams of gallic acid equivalent per gram of dry matter (mg GAE/100 g DW). TFC was determined according to the method outlined by Al-Duais et al. [31] with slight modifications. A total of 75 μL of 5% sodium nitrite solution was mixed with 250 μL of the MeOH extract from each sample, and the mixture was vortexed. Following a 5 min incubation at room temperature, 150 μL of 10% aluminum chloride was added, and the mixture was vortexed. The reaction mixture was then incubated for 6 min at room temperature, followed by the addition of 500 μL of 1 N NaOH, and absorbance was measured at 510 nm using a spectrophotometer (Biochrom; Libra 22 UV/Visible Spectrophotometer; 177 Holliston, MA, USA). The TFC was determined using a calibration curve for quercetin, and the results were expressed as mg quercetin equivalent (QE) per 100 g of flour.

### 2.6. Physicochemical and Functional Characterization

Chemical analyses were performed in triplicate according to the guidelines of the Association of Official Analytical Chemists. Moisture content was determined from a homogenized sample by measuring the weight loss that occurred during desiccation, which continued until a constant weight was obtained. Ash content was determined using the gravimetric method. The lipid content was determined by Soxhlet extraction. Protein content was determined with the Kjeldahl method, and total dietary fiber was determined through the enzymatic gravimetric method [32,33]. The antioxidant activity of the concentrated UAE extracts, redissolved in 70% ethanol (*v*/*v*), was evaluated using a DPPH radical scavenging assay [34]. A volume of 100 μL of the sample solution was mixed with 3.9 mL of freshly prepared 0.1 mol/L DPPH radical solution. After 30 min of reaction in the dark at room temperature, the absorbance was measured at 515 nm using a JENWAY spectrophotometer (Model 6505 UV-Vis, Great Dunmow, UK).

### 2.7. Phytic Acid Content

For the determination of phytic acid content, 2 g of teff flour passing through a 500 µm sieve was mixed with 40 mL of 0.4 M HCl/5% Na_2_SO_4_ extraction solution and allowed to stand for 90 min with occasional stirring. After filtration, 25 mL of filtrate was mixed with 20 mL of 0.4 M HCl/5% Na_2_SO_4_ solution, 20 mL of ferric chloride solution, and 20 mL of sulfosalicylic acid. The mixture was boiled in a closed tube in a water bath for 15 min, then cooled under a running water jet. The ferric precipitate was made and isolated by filtration. From the filtrate, 25 mL was taken and placed in an Erlenmeyer flask. Then, 175 mL of distilled water was added, after which the pH was adjusted to 2.5 ± 0.5 with glycol salt solution. The resulting mixture was heated to 70 °C, and the excess of Fe^3+^ that had not precipitated with phytic acid was titrated with 0.01 M EDTA solution until the color turned bright yellow [35].

### 2.8. High-Performance Liquid Chromatography

Polyphenolic compounds in the extracts were identified at wavelengths of 280 nm and 320 nm, following the method described by Mërtiri et al. [36] using an Agilent 1200 HPLC system. This system features a diode-array detector (DAD) (Agilent Technologies, Santa Clara, CA, USA) equipped with a degasser, a quaternary pump system, an autosampler, a column, and a multi-wavelength detector. Compounds were separated using a BDS Hypersil C18 column (150 × 4.6 mm, 5 μm) under the following conditions: 30 °C, 10 μL injection volume, and 1 mL/min flow rate. The mobile phases consisted of (A) 100% methanol and (B) 10% formic acid. The linear solvent gradient consisted of the following: 0–20 min, 9% A–91% B; from 20 to 30 min, 35% A–65% B; from 30 to 40 min, 50% A–50% B, from 40 to 45 min, 9% A–91% B. Results are expressed as average values for duplicate measurements ± standard deviation, in micrograms per gram extract (μg/g extract).

### 2.9. Optimization with the Desirability Function

To optimize the extraction of bioactive compounds from teff, Derringer’s desirability function was applied in Minitab 20 (Minitab LLC, State College, PA, USA) to optimize multiple responses simultaneously. For each response, an individual desirability value, ranging from 0 (completely undesirable) to 1 (fully desirable), was calculated according to the selected optimization goal (maximize). With these individual desirability values, the overall desirability (D) can be obtained. The overall desirability function D is defined as the weighted geometric average of the individual desirability (*d_i_*) according to the following equation,

(2) $$D=\sqrt[{m}]{d_{1\;\;}d_{2\;\ldots \ldots .\;\;}d_{m\;\;}}$$

where m is the number of responses studied in the optimization process. Thus, the simultaneous optimization process is reduced to finding the levels of factors that demonstrate the maximum overall desirability. The optimum extraction conditions were identified by maximizing the composite desirability (D), with equal importance assigned to all responses [37,38]. This method enabled the identification of ideal conditions that simultaneously enhanced the concentration of phenolic compounds and the antioxidant potential of teff extracts [38,39].

### 2.10. Phytic Acid Degradation

For the thermal degradation assay, 2 g of teff flour was extracted with 40 mL of 0.4 M HCl/5% Na_2_SO_4_ for 90 min with intermittent shaking. After filtration, 25 mL of extract was heated in a thermostatic water bath at 50 °C (0–90 min), 70 °C (0–90 min), or 90 °C (0–180 min), and then rapidly cooled in an ice bath. Subsequently, 25 mL of cooled extract was mixed with 20 mL of 0.4 M HCl/5% Na_2_SO_4_ solution, 20 mL of FeCl_3_ solution (0.2 M in 0.16 M HCl), and 20 mL of 20% sulfosalicylic acid solution, boiled for 15 min in a water bath, cooled under running water, and filtered to separate the precipitate. An aliquot of 25 mL of the filtered extract was then diluted with 175 mL of distilled water, and the pH was adjusted to 2.5 ± 0.5 using glycine–saline buffer. The solution was heated to 70 °C and titrated with 0.01 M EDTA while hot, using sulfosalicylic acid as an indicator until a yellow endpoint was reached [40,41].

### 2.11. Kinetic Data Analysis

The degradation kinetics of the phenolic and flavonoid contents of teff and the antioxidant capacity are modeled as first-order kinetics using the first-order model equation (Equation (2)) to describe this reaction and the degradation rate constants with respect to heating time:

*C*/*C*_0_ = *e*^−*kt*^ (3)

where C represents the variable being predicted by the model, C_0_ is the initial value of that variable, t is the amount of time that the sample has been subjected to heat, and k is the rate constant (1/min) at a particular temperature (T).

Thereby, the time required for the reaction to reach 50% completion (i.e., the half-life (t_1/2_) of this reaction) can be calculated, assuming first-order kinetics, using Equation (3):

*t*_1/2_ = −*ln* (0.5)/*k* (4)

where k is the degradation rate constant. In the present study, the Arrhenius model was used to describe the effect of temperature on the rate constant of the degradation reaction, as shown by Equation (4):

*k* = *k*_*ref* × *exp* [−*Ea*/(*R* × *T*)] (5)

where Ea refers to activation energy (kJ/mol), k ref refers to the reaction rate constant, R refers to the universal gas constant, and T is the absolute temperature of the reaction being modeled or predicted.

The kinetic parameter Ea was estimated through linear regression of the natural logarithm of the degradation rate constant against the reciprocal of absolute temperature. The accuracy of these equations was assessed by visually inspecting the linearity of the graphs, the regression coefficients, and the residuals.

### 2.12. Microbiological Analysis

The prebiotic potential of teff flour extract was assessed by preparing anaerobic batch cultures and inoculating them with freeze-dried strains of *Lactobacillus plantarum* MIUG BL21, recovered and maintained at 6 °C until use, at an initial concentration of 10^8^ CFU/mL. Teff flour extract was first dried at 50 °C, and then 1 mg, 2.5 mg, and 5 mg of each of the three concentrations were dissolved in 90 mL of sterile Man, Rogosa and Sharpe (MRS) broth. The mixtures were thoroughly shaken and incubated at 37 °C for 24 h under aerobic conditions. Before inoculation, the media were cooled in a biosafety cabinet. The samples were inoculated with 2% (*v*/*v*) freeze-dried strains of *Lactobacillus plantarum* MIUG BL 21, the inoculum having an optical density of 2 at λ = 600 nm, and incubated at 37 °C for 72 h and then stored at 4 °C for 21 days, the indirect bacterial count being determined initially and after 7, 14, and 21 days. A sterile saline solution (0.9% *w*/*v* NaCl) was prepared and autoclaved at 121 °C for 15 min. To obtain viable counts, 1 mL of the sample was mixed with 9 mL of saline solution. Appropriate dilutions of aliquots were plated on MRS agar (Carl Roth GmbH, Karlsruhe, Germany). Plates were incubated at 37 °C for 72 h under aerobic conditions, and results were reported as log CFU/mL [42].

### 2.13. Statistical Methods

Using Minitab 20 (2019, Minitab, Inc., State College, PA, USA), Box–Behnken experimental designs and all response surface methodology analyses were performed. All results were presented as the average of three repetitions ± Standard Deviation (SD). Analysis of variance (ANOVA) was performed for all regression equations to assess their level of significance and determine whether they could be used. A *p*-value < 0.05 was defined as statistically significant. Optimal extraction conditions were established using the response optimizer function. The Pearson correlation coefficient was used to evaluate the relationships among TPC, TFC, and DPPH scavenging activity.

## 3. Results and Discussion

### 3.1. Effect of Extraction Parameters

**Figure 1:**
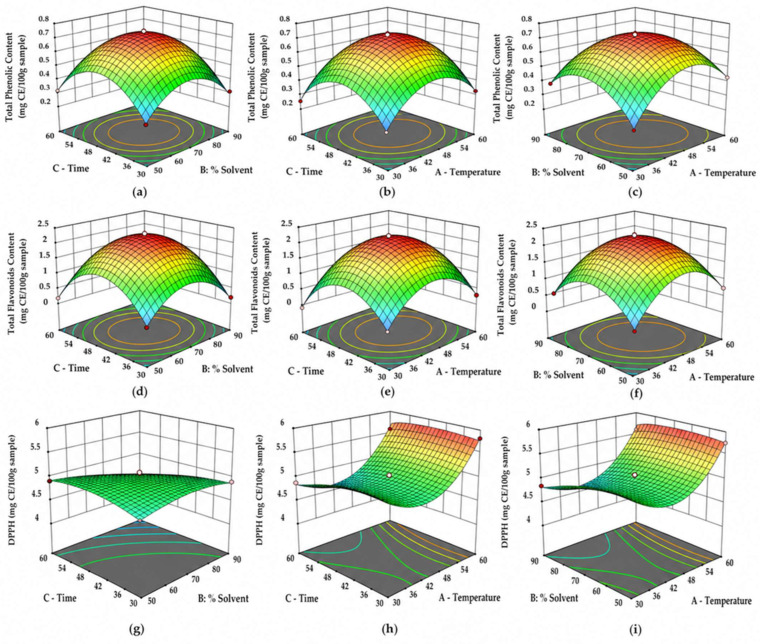
Response surface plots showing the interactive effects of total phenol (**a**–**c**), total flavonoid (**d**–**f**), and DPPH (**g**–**h**) on the extraction yield of total phenol, flavonoid, and DPPH contents.

The response surface plots (Figure 1) showed the influence of independent variables such as temperature, solvent concentration, and extraction time on the extraction of total phenolic content, total flavonoid content, and antioxidant activity. Moderate temperature (36.9 °C), ethanol concentration (50%), and time (60 min) resulted in the highest yields of phenolic and flavonoid compounds, as well as antioxidant activity. These results agree with previous findings that mixed solvents, particularly ethanol–water systems, enhance polyphenol solubility by modulating polarity, thereby facilitating the extraction of both polar and moderately polar compounds [43,44]. These conditions were determined as optimal based on the Box–Behnken design and the second-order polynomial regression model, indicating a strong fit with high R^2^ values for TPC and TFC. In general, the response surfaces indicated that a medium ethanol concentration (70%) yielded significantly higher results across all three responses. As observed in previous studies, ethanol–water mixtures exhibit an optimal polarity range for the efficient extraction of both polar and moderately non-polar phytochemicals, including polyphenols and flavonoids [45,46]. Higher ethanol concentrations tended to reduce efficiency due to cellular dehydration, which hinders the diffusion of compounds from plant matrices [47,48]. The extraction time showed a bell-shaped pattern, with initial increases in duration enhancing extraction, but excessive durations (>45 min) led to degradation of thermolabile phenolics, reducing TPC and DPPH activity, as also observed by Feki et al. [45] and Väisänen et al. [49]. Similarly, TFC and DPPH radical scavenging followed similar trends, indicating the direct relationship between phenolic and flavonoid content and antioxidant activity [43,46]. Moreover, the DPPH antioxidant activity increased proportionally with TPC and TFC levels, reaffirming that phenolic and flavonoid compounds are key contributors to the antioxidant properties of teff extracts. However, co-extracted bioactive substances with antioxidant activity, like polysaccharides or minor pigments, might also contribute to DPPH activity, as Väisänen et al. suggest [49].

A second-order polynomial model was used to statistically analyze the efficiency of phenolic compound extraction from teff. The ANOVA (Table 2) confirmed the model’s significance, with an F-value of 60.97 and a *p*-value < 0.0001, indicating that it accurately represents the experimental data. This result is consistent with previous studies that optimized phenolic extraction from various plant matrices using response surface methodology [50]. Temperature, % solvent, and extraction time were all statistically significant, indicating that each independently influences the yield of extracted phenolic compounds. Temperature significantly impacted the F value (152.63), aligning with the literature, which shows that temperature increases cell wall permeability and phenolic solubility [51]. The extraction parameters significantly affected the results, with combined effects among these variables boosting phenolic yield. Notably, the association between temperature and time was significant, supporting the idea that optimal extraction mainly depends on thermal and temporal factors [52]. The quadratic terms (A^2^, B^2^, and C^2^) showed very high F-values and strongly significant *p*-values, indicating curvature and confirming the importance of a quadratic model for predicting phenol extraction yields. The model’s robustness was validated by a non-significant lack of fit (0.9662) and excellent indicators of R^2^ (0.9910), adjusted R^2^ (0.9747), predicted R^2^ (0.9667), and adequate precision (26.86), exceeding the desirable threshold of 4. The low standard deviation and coefficient of variation (3.79%) highlight the accuracy and consistency of the experimental data. These results agree with early studies on phenol extraction optimization from agro-based materials, which reported similar model significance and factor effects when using RSM [53].

### 3.2. Optimization of Extraction Conditions

**Figure 2:**
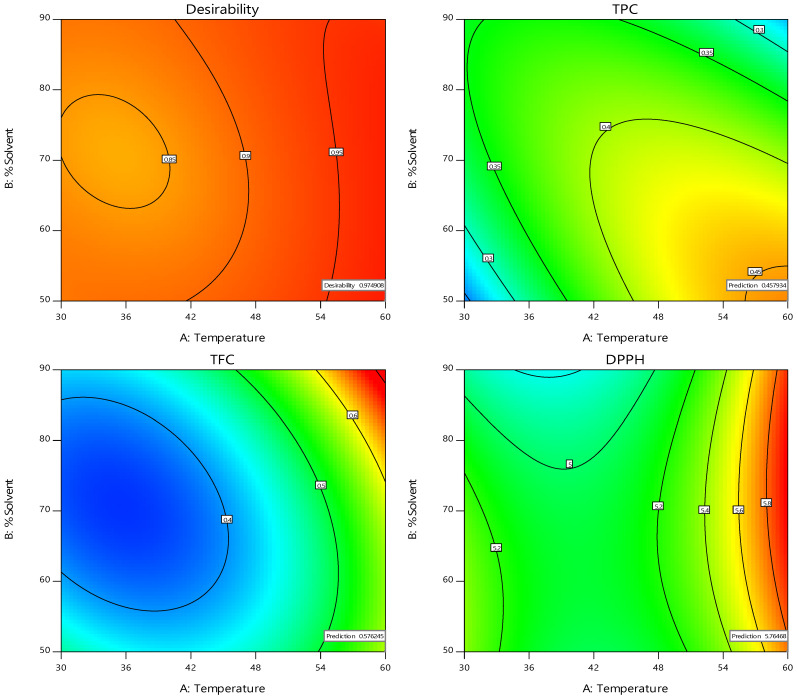
The contour plot that shows the overall desirability, total phenolic content, total flavonoid content, and DPPH radical scavenging activity.

Using a desirability approach, all three responses were optimized to maximize total phenolic content (TPC), total flavonoid content (TFC), and antioxidant activity (DPPH) (Figure 2). Multiple criteria were developed and used to create the desirability function based on the Response Surface Model and the practical importance of each response. Each extraction condition had a designated response and together represented a successful combination of maximum phenolic yield, flavonoid content, and antioxidant capacity. The TPC, TFC, and DPPH values were maximized within their respective factor ranges under the optimal conditions of the study, demonstrating the feasibility of the optimization approach. The optimal extraction conditions were consistent with the experimental design, indicating that the boundaries did not influence them.

Throughout the experimental runs, TPC values ranged from 20.9 to 49.6 mg GAE/100 g DW (Table 3). The maximum TPC value was obtained at 50% solvent, 60 °C, and 45 min, indicating that relatively high temperature and moderate solvent concentration promoted the release of phenolic compounds. However, the lowest TPC values were observed at low temperature and short extraction times (20.9 mg GAE/100 g DW at 30 °C and 60 min), indicating inefficiency in solubilizing bound phenolics under mild conditions. Similar patterns have been observed in cereal grains, where higher temperatures increase cell wall permeability and enhance solvent penetration without causing significant thermal degradation when applied within a controlled range [19,54]. The central point runs at 45 °C, 70% solvent, 45 min, and consistently shows TPC values between 42.1 and 45.9 mg GAE/100 g DW, verifying good experimental reproducibility. These responses also revealed that excessively high solvent concentration (greater than 70%) does not consistently improve phenolic extraction. At elevated ethanol concentrations, diminished polarity may reduce the solubility of certain hydrophilic phenolics, resulting in lower yields, a phenomenon also observed in teff and other small grains [55,56].

The Total Flavonoid Content (TFC) values ranged from 35.1 to 67.9 mg CE/100 g DW, with the highest TFC observed at 60 °C, 90% solvent, and 45 min. This indicates that flavonoids in teff are more responsive to increased solvent concentration compared to total phenolics, likely because many flavonoids exhibit greater solubility in less polar solvent systems. Similar results were observed in barley, millet, and colored rice extracts, where optimal TFC recovery was obtained at higher ethanol levels [57,58]. Although extraction beyond optimal polarity may result in co-extraction of non-phenolic compounds, potentially diluting the apparent flavonoid fraction [59].

The antioxidant activity of teff varied from 4.36 to 5.94 mMol Trolox/g DW. The higher radical-scavenging capacity, 5.79 mMol Trolox/g DW, was achieved at 50% solvent, 30 °C, and 45 min, whereas the minimum values were observed under conditions that yielded both lower TPC and TFC. While TPC and DPPH showed mostly positive correlations, some deviations indicate that antioxidant capacity is not solely determined by phenolic concentration but also by phenolic structure and the presence of other antioxidant constituents, such as flavonoids and organic acids [60,61]. Comparable observations have been reported in sorghum and millet extracts, where antioxidant responses varied despite similar phenolic totals [62,63]. The optimized teff extracts demonstrated phenolic contents similar to those of finger millet, barley, and red rice and were higher than values commonly reported for wheat and maize [54,57].

### 3.3. Proximate Composition of Teff Extract

The data in Table 4 indicate the proximate composition of white, mixed, and red teff flour. Moisture content ranged from 12.93 g/100 g in red teff to 14.47 g/100 g in mixed teff. The observed moisture content across different teff varieties matches previous research, which reports moisture levels in teff grains to range from 8.33% to 10.53%. The slightly higher moisture content in mixed teff (14.47 g/100 g) may result from varietal differences and environmental conditions during growth and storage. Lower moisture content, such as in red teff (12.93 g/100 g), helps improve storage stability and shelf life by lowering the risk of microbial growth [64]. The protein content across the teff varieties in this study (6.75 ± 2.17–8.33 ± 0.18 g/100 g) aligns with the range reported in the recent literature, which states teff’s protein content between 8.7% and 11% [65,66]. Teff’s protein is notable for its high lysine content, an essential amino acid often limited in other cereals, enhancing its nutritional value. The slightly higher protein content in red teff may be beneficial for populations relying on plant-based protein sources.

Lipid content was highest in red teff (3.06 g/100 g) and lowest in white teff (2.68 g/100 g). The observed lipid content, especially the higher value in red teff (3.06 ± 0.07 g/100 g), falls within the reported range for teff grains (2.75% to 3.12%) [64,67]. Teff lipids are high in unsaturated fatty acids, including linoleic and oleic acids, which support cardiovascular health benefits. Differences in lipid levels among teff varieties may affect their energy content and their suitability for specific dietary needs.

Ash content, which indicates total mineral content, was highest in mixed teff (2.77 ± 0.007 g/100 g), pointing to a richer mineral profile. This matches previous studies reporting ash content in teff ranging from 2.33% to 6.39% [64]. Teff is known for its high mineral content, especially calcium and iron, which are important for bone health and the prevention of anemia. Fiber content was highest in white teff (4.20 ± 0.90 g/100 g), followed by mixed (4.06 ± 0.52 g/100 g) and white teff (3.97 ± 0.977 g/100 g). These values support teff’s role in promoting gut health and regulating blood glucose levels. High dietary fiber intake is linked to reduced risks of cardiovascular disease and improved metabolic health [65]. Red teff might be preferred when emphasizing dietary fiber intake.

### 3.4. Effect of Processing on the Antinutritional Compounds

#### 3.4.1. Phytate Content

Phytic acid content ranged from 0.09 g/100 g in white teff to 0.11 g/100 g in mixed teff. Red teff had the lowest phytic acid content at 0.09 ± 0.004 g/100 g, while mixed teff had the highest at 0.11 ± 0.00079 g/100 g. Although phytic acid is known as an antinutrient due to its mineral-binding properties, it also exhibits antioxidant activity and may help protect against certain diseases. Processing methods such as fermentation and heat treatment can reduce phytic acid levels, thereby improving mineral absorption in teff-based foods [68].

#### 3.4.2. Phytate

Kinetic models serve as practical, economical tools for assessing food quality and safety under various processing conditions. They enable the prediction of nutrient degradation and optimization of processing parameters by providing insight into reaction order, rate constants, and activation energy [62,63]. In this study, phytic acid degradation in teff was evaluated during isothermal heating at 50 °C, 70 °C, and 90 °C, using a first-order kinetic model, ln(C/C_0_) = −kt, and t_1/2_ = 0.693/k.

#### 3.4.3. First-Order Degradation Kinetics of Phytic Acid

**Figure 3:**
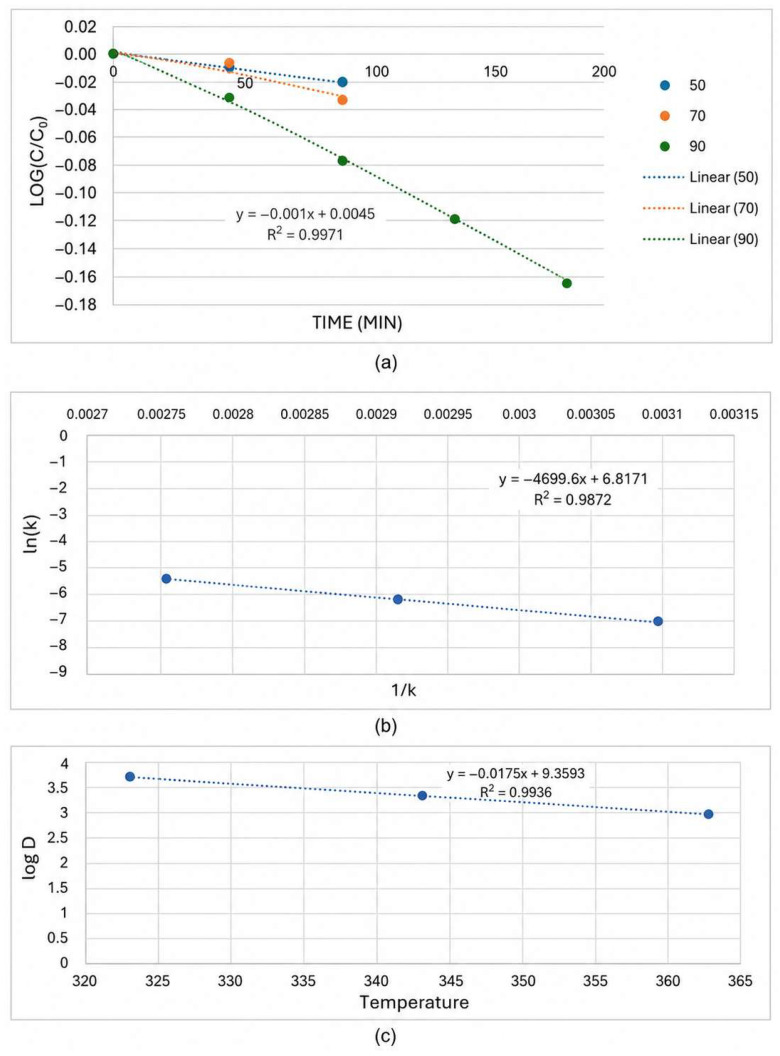
Thermal degradation of phytic acid at different temperatures and times: (**a**) first-order kinetic plots of phytic acid degradation at 50 °C, 70 °C, and 90 °C, showing the linear relationship between ln(C/C0) and time; (**b**) Arrhenius plot illustrating the dependence of the degradation rate constant (k) on the reciprocal of absolute temperature (1/T); (**c**) plot of log D versus temperature, demonstrating the effect of temperature on the degradation process.

Linear regression analysis confirmed that phytic acid degradation followed first-order kinetics at all three temperatures (Table 5). As shown in Figure 3 the degradation rate constant (k) increased with temperature, confirming the reaction’s temperature dependence. At 50 °C, the rate constant was 0.00046 min^−1^ with R^2^ of 1.000, resulting in a half-life (t_1/2_) of 1504.55 min. At 70 °C, the rate increased to 0.00092 min^−1^ with a corresponding t_1/2_ of 752.3 min with R^2^ of 0.8911, and further increased at 90 °C to 0.0023 min^−1^, with a reduced half-life of 300.91 min with R^2^ of 0.9971.

The thermal degradation kinetics of phytic acid in teff were investigated at 50 °C, 70 °C, and 90 °C, revealing a temperature-dependent degradation pattern (Table 6). The data conformed to first-order kinetics, as evidenced by the linear relationship between ln(Ct/C_0_) and time, with a high correlation coefficient (R^2^ = 0.99). The calculated rate constants (k) increased with temperature, indicating accelerated degradation at higher temperatures. The Arrhenius plot (ln k vs. 1/T) demonstrated a linear trend with R^2^ of 0.98, allowing for the estimation of the activation energy (Ea) for phytic acid degradation. The calculated Ea was approximately 39.07 kJ/mol, aligning with values reported for thermal degradation of antinutrients in cereals.

These findings are consistent with recent studies highlighting the impact of thermal processing on phytic acid content in cereals. For instance, Li et al. [69] reported that elevated storage temperatures accelerate the degradation of phytic acid in stored foods. Similarly, heat-processing methods have been shown to alter the starch matrix in cereals, thereby affecting mineral bioavailability. In the context of teff, traditional fermentation processes, such as those used in injera preparation, have been shown to reduce phytic acid content significantly. Fischer et al. [5] observed varying degrees of phytic acid degradation during spontaneous fermentation of teff dough, ranging from 42% to 80%. These reductions are attributed to the activity of naturally occurring lactic acid bacteria that degrade phytic acid. Reducing phytic acid through thermal processing has important nutritional implications. Phytic acid is known to chelate essential minerals like iron and zinc, reducing their bioavailability. Therefore, its degradation can enhance the nutritional quality of teff-based foods by improving mineral absorption. This is particularly relevant in populations where teff is a staple food and mineral deficiencies are prevalent.

### 3.5. HPLC Characterization of Phenolic Compounds in the Extract

**Figure 4:**
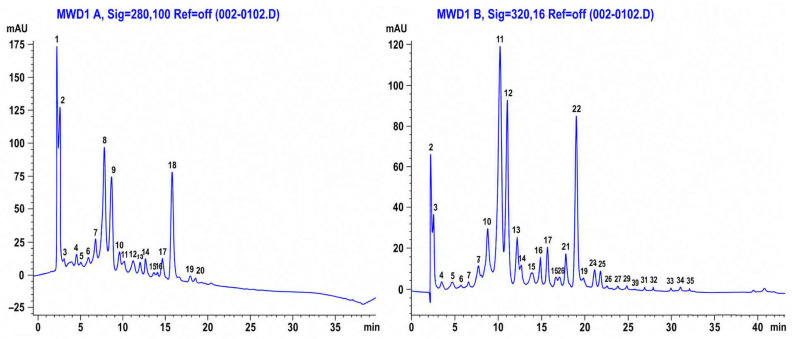
Phenolic compounds profile extract (**Left**) 280 nm: 1—Unidentified compound; 2—epigallocatechin; 3—unidentified compound; 4—caffeic acid; 5—epicatechin; 6—syringic acid; 7—p-coumaric acid; 8—unidentified compound; 9—ferulic acid; 10–13—unidentified compounds; 14—naringin; 15—rutin trihydrate; 16—unidentified compound; 17—hesperidin; 18–20—unidentified compounds. (**Right**) 320 nm: 2—Unidentified compounds; 3—epigallocatechin; 4–6—unidentified compounds; 7—epicatechin; 8—syringic acid; 9—caffeine; 10—p-coumaric acid; 11—unidentified compound; 12—ferulic acid; 13–17—unidentified compounds; 19—rutin trihydrate; 20—unidentified compound; 21—hesperidin; 22—unidentified compound; 23—cinnamic acid; 24–26—unidentified compounds; 27—quercetin; 29—unidentified compounds; 30—luteolin; 31–34—unidentified compounds; 35—apigenin.

The results of the HPLC identification and quantification are shown in Table 7, while the typical chromatograms are presented in Figure 4. Analysis of teff (*Eragrostis tef*) extracts at 280 nm and 320 nm revealed a diverse range of phenolic compounds. Thirty-five different peaks were identified, representing various phenolic acids and flavonoids. In the UAE, 13 bioactive compounds were identified, among which were four polyphenolic acids (caffeic acid, vanillic acid, p-coumaric acid, and ferulic acid) and nine flavonoids (epigallocatechin, epicatechin, quercetin, quercetin dihydrate, hesperidin, rutin trihydrate, naringin, apigenin, and luteolin).

The most prevalent compound was epigallocatechin, quantified at 2960.30 µg/g during extraction. This level is relatively high for a cereal grain. In comparison, recent studies on barley have reported catechin levels ranging from 0 to 21.85 µg/g, depending on the barley type and processing methods [69]. The epicatechin content was 113.55 µg/g, within the typical range of 4–35 µg/g reported for sorghum and millet varieties [70]. The main phenolic acid was ferulic acid, at 41.79 µg/g, consistent with its established dominance in cereal matrices. However, this level remains lower than the concentrations found in wheat bran, which can range from 1.36 to 2.51 mg/g depending on the wheat variety and processing method [71]. Other flavonoids found included hesperidin (163.64 µg/g), naringin (40.41 µg/g), quercetin (11.43 µg/g), and luteolin (9.21 µg/g). These compounds are more commonly associated with citrus fruits and leafy vegetables, and their presence in teff is relatively rare among cereal grains. For comparison, oats have shown flavonoid concentrations ranging from 1.7 to 31.3 mg/g, depending on genotype [72], and maize displays a wide range of flavonoid metabolites that vary throughout development [73]. Vanillic acid and cinnamic acid weren’t detected in the teff extracts. This might be because their levels are too low to detect, or because they broke down slightly during extraction and processing. In comparison to other cereals such as quinoa and amaranth, which have ferulic acid-glucoside levels of about 132–161 mg/kg and total flavonoids ranging from 36.2 to 144.3 mg/100 g [74,75], teff appears to exhibit a distinct phenolic fingerprint. Its relatively high levels of catechins and flavonoids suggest that teff may offer unique nutritional and functional benefits among cereal grains.

### 3.6. Prebiotic Activity of Teff

The viability of probiotic microorganisms in teff-supplemented media stored at 4 °C over 21 days was assessed. As shown in Table 8, the survival of probiotic bacteria (*Lactobacillus plantarum* MIUG BL21) varied significantly depending on the concentration of teff extract. The 5 mg/mL extract demonstrated the highest probiotic viability, with counts decreasing from 4721 ± 0.049 log CFU/mL on day 0 to 2349 ± 0.048 log CFU/mL by day 21. This sustained viability suggests that teff extract at this concentration serves as an effective prebiotic matrix supporting long-term microbial stability. In contrast, the control showed a steep decline in viability, from 3535 ± 0.077 to 0.150 ± 0.150 log CFU/mL over 21 days, underscoring the inadequacy of the base medium for maintaining probiotic populations. The 2.5 mg/mL extract group retained relatively better viability, decreasing from 3396 ± 0.095 to 0.500 ± 0.198 log CFU/mL. Meanwhile, the 1 mg/mL group exhibited limited protective effects; after a small drop during the first two weeks, the probiotic count fell below detectable levels by day 21.

These results suggest a clear dose-dependent effect of teff extract on probiotic viability, consistent with findings from similar studies involving cereal-based prebiotics [76,77,78]. The increased stability at higher concentrations probably results from teff’s rich supply of fermentable fibers, resistant starch, and oligosaccharides, which serve as selective substrates for beneficial bacteria like *Lactobacillus* spp. [76,79]. Furthermore, the presence of bioactive compounds, mainly phenolic acids such as ferulic acid, gallic acid, and catechins, may contribute to microbial stability by exerting antioxidant and protective effects [24,80]. These phenolics can modulate the gut environment, enhancing both bacterial adhesion and resistance to oxidative stress [81,82]. These results were in line with earlier findings that teff-enriched media enhance probiotic survival during refrigerated storage [76]. The significant decline in the control sample underscores the importance of functional bioactive ingredients, such as those in teff, to support viable probiotic delivery in food systems [83].

## 4. Conclusions

This study highlights the multifaceted nutritional and functional value of *Eragrostis tef*, demonstrating its potential as a promising ingredient in health-oriented food applications. The extraction of bioactive-enriched extract and the antioxidant activity of teff were evaluated using an ultrasound-assisted extraction method and high-performance liquid chromatography, respectively. Optimization was performed using a Box–Behnken Design, and RSM allowed testing different values of the independent variables to achieve maximum responses. Therefore, the optimum parameters were established as 45 ◦C, 50% ethanol, and 60 min. Prebiotic activity was assessed through culturing the beneficial bacteria for 21 days under refrigeration (4 °C). Teff extracts, particularly at 5 mg/mL, maintained probiotic viability during refrigerated storage compared with the control, suggesting their potential use as functional food matrices. Moreover, thermal degradation of phytic acid revealed that controlled heating significantly reduces antinutritional factors, following first-order kinetics. This indicates that mild thermal processing can enhance mineral bioavailability while preserving key phytochemicals. Teff shows great potential to develop into functional foods that support antioxidant defense, encourage a healthy gut microbiome, and enhance nutrient absorption. These results suggest promising potential for the use of teff extracts in additional food applications.

## Figures and Tables

**Table 1:** Range and levels of independent variables for UAE optimization.

Variables	Code	Code Levels		
		Lower	Central	Higher
Ethanol concentration (%, *v*/*v*)	A	50	70	90
Extraction time (min)	B	30	45	60
Temperature (°C)	C	30	45	60

**Table 2:** ANOVA of quadratic response surface model for total phenols and flavonoids in teff.

Total Polyphenol Content						Total Flavonoid Content				
Variables	Sum of Squares	Df	Mean Square	F-Value	*p*-Value	Sum of Squares	Df	Mean Square	F-Value	*p*-Value
Model	0.0951	9	0.0106	60.97	0.0001	0.1379	9	0.0153	93.09	<0.0001
A	0.0264	1	0.0264	152.63	<0.0001	0.0745	1	0.0745	452.78	<0.0001
B	0.0013	1	0.0013	7.50	0.0408	0.0017	1	0.0017	10.22	0.0241
C	0.0003	1	0.0003	1.66	0.2538	0.0098	1	0.0098	59.56	0.0006
AB	0.0222	1	0.0222	128.11	<0.0001	0.0088	1	0.0088	53.70	0.0007
AC	0.0083	1	0.0083	47.78	0.0010	0.0005	1	0.0005	2.94	0.1470
BC	0.0016	1	0.0016	9.23	0.0288	0.0013	1	0.0013	7.88	0.0377
A^2^	0.0074	1	0.0074	42.67	0.0013	0.0243	1	0.0243	147.84	<0.0001
B^2^	0.0053	1	0.0053	30.36	0.0027	0.0154	1	0.0154	93.84	0.0002
C^2^	0.0265	1	0.0265	153.03	<0.0001	0.0072	1	0.0072	43.78	0.0012
Residual	0.0009	5	0.0002			0.0008	5	0.0002		
Lack of Fit	0.0001	3	0.0000	0.0777	0.9662	0.0003	3	0.0001	0.3990	0.7709
Pure Error	0.0008	2	0.0004			0.0005	2	0.0003		
Cor Total	0.0960	14				0.1387	14			
Std. Dev.	0.0132					0.0128				
C.V. %	3.79					2.73				
*R^2^	0.9910					0.9941				
*R^2^. adj	0.9747					0.9834				
Adeq. Precision	26.86					29.01				

A: Temperature; B: % solvent; C: extraction time; *R^2^ and *R^2^. adj are calculated for the reduced model. C.V. % coefficient of variation.

**Table 3:** Experimental design generated using the Box–Behnken Design with dependent and independent variables studied to optimize extractions (ethanol/water, *v*/*v*).

Run	Temperature, °C	Solvent, %	Time, min	TPC, mg GAE/100 g DW	TFC, mg CE/100 g DW	DPPH, mMol Trolox/g DW
1	60	70	60	42.1 ± 0.001	53.7 ± 0.003	5.63 ± 0.018
2	30	70	30	28.5 ± 0.006	42.7 ± 0.021	5.22 ± 0.006
3	60	70	30	31.5 ± 0.019	63 ± 0.004	5.94 ± 0.205
4	30	70	60	20.9 ± 0.023	37.8 ± 0.003	4.96 ± 0.040
5	60	50	45	49.6 ± 0.028	55.3 ± 0.004	5.79 ± 0.132
6	45	90	60	32.6 ± 0.004	43.7 ± 0.043	4.36 ± 0.064
7	30	90	45	36.2 ± 0.014	38 ± 0.000	4.82 ± 0.101
8	45	90	30	27.7 ± 0.020	54.2 ± 0.037	4.92 ± 0.291
9	30	50	45	23.8 ± 0.002	44.2 ± 0.009	5.4 ± 0.106
10	60	90	45	32.2 ± 0.004	67.9 ± 0.009	5.78 ± 0.018
11	45	50	60	31.2 ± 0.004	44.7 ± 0.042	4.94 ± 0.226
12	45	70	45	42.1 ± 0.002	38.3 ± 0.039	5.09 ± 0.021
13	45	70	45	45.9 ± 0.005	35.1 ± 0.008	5.06 ± 0.047
14	45	70	45	43.1 ± 0.010	36.9 ± 0.021	5.077 ± 0.021
15	45	50	30	34.3 ± 0.004	48 ± 0.009	4.95 ± 0.024

**Table 4:** Proximate composition of teff.

Components (g/100 g)	White Teff	Mixed Teff	Red Teff
Moisture	13.71 ± 0.020	14.39 ± 0.100	12.80 ± 0.180
Protein	7.7 ± 0.730	6.75 ± 2.170	8.33 ± 0.180
Lipid	2.68 ± 0.090	3.02 ± 0.026	3.06 ± 0.070
Ash	2.67 ± 0.001	2.77 ± 0.007	2.12 ± 0.290
Fiber	4.20 ± 0.900	4.06 ± 0.520	3.97 ± 0.980

All values are means of triplicate determinations ± standard deviations.

**Table 5:** Degradation kinetics of phytic acid.

Temperature (°C)	T (K)	Rate Constant (k, min^−1^)	ln(k)	Half-Life (t_1/2_, min)	R^2^	Ea (KJ/Mol)
50	323	0.00046	−7.68	1504.60	1.00	39.07
70	343	0.00092	−6.99	752.30	0.89	
90	363	0.00230	−6.07	300.91	0.99	

**Table 6:** ANOVA and Regression Coefficients for Phytic Acid Degradation in Teff.

	Coefficients	Standard Error	t Stat	*p*-Value	Lower 95%	Upper 95%	
Intercept	6.817	1.567	4.34	0.14	−13.10	26.73	
X Variable 1	−4699.59	535.8	−8.77	0.072	−11,508.153	2108.981	
	−39.077	4.456					
ANOVA and Regression Statistics							
	df	SS	MS	F	Significance F	Multiple R	0.99
Regression	1	1.28	1.28	76.9	0.07	R Square	0.98
Residual	1	0.0167	0.0167			Adjusted R-Square	0.97
Total	2	1.3034				Standard Error	0.12
						Observations	3

**Table 7:** HPLC profile of bioactive compounds in teff extracts.

No.	Identified Compound	Concentration (μg/g)
1	Caffeic acid	9.724
2	Vanillic acid	n.d.
3	Syringic acid	10.246
4	P-coumaric acid	13.758
5	Ferulic acid	41.791
6	Cinnamic acid	n.d.
7	Luteolin	9.208
8	Epicatechin	113.546
9	Epigallocatechin	2960.298
10	Naringin	40.407
11	Rutin trihydrate	8.089
12	Hesperidin	163.638
13	Apigenin	7.478
14	Quercetin	11.433
15	Quercetin dihydrate	12.271
16	Caffeine	n.d.

n.d.—Not detected.

**Table 8:** Prebiotic effect of teff extracts upon *L. plantarum*.

Concentration of Extract (mg/mL)	Log CFU/mL After Storage at 4 °C, Days			
	0	7	14	21
Control	3.535 ± 0.077	2.781 ± 0.003	0.772 ± 0.073	0.150 ± 0.150
1 mg/mL	2.326 ± 0.150	2.209 ± 0.033	1.020 ± 0.020	0.000± 0.000
2.5 mg/mL	3.396 ± 0.095	3.481 ± 0.086	1.238 ± 0.238	0.500 ± 0.198
5 mg/mL	4.721 ± 0.049	4.079 ± 0.124	3.190 ± 0.110	2.349 ± 0.048

## Data Availability

The original contributions presented in this study are included in the article. Further inquiries can be directed to the corresponding author.
